# Blood Beta-Amyloid and Tau in Down Syndrome: A Comparison with Alzheimer’s Disease

**DOI:** 10.3389/fnagi.2016.00316

**Published:** 2017-01-17

**Authors:** Ni-Chung Lee, Shieh-Yueh Yang, Jen-Jie Chieh, Po-Tsang Huang, Lih-Maan Chang, Yen-Nan Chiu, Ai-Chiu Huang, Yin-Hsiu Chien, Wuh-Liang Hwu, Ming-Jang Chiu

**Affiliations:** ^1^Department of Pediatrics, National Taiwan University Hospital, College of Medicine, National Taiwan UniversityTaipei, Taiwan; ^2^Department of Medical Genetics, National Taiwan University Hospital, College of Medicine, National Taiwan UniversityTaipei, Taiwan; ^3^MagQu Co., Ltd.,New Taipei City, Taiwan; ^4^Institute of Electro-Optical Science and Technology, National Taiwan Normal UniversityTaipei, Taiwan; ^5^Department of Clinical Psychology Center, National Taiwan University Hospital, College of Medicine, National Taiwan UniversityTaipei, Taiwan; ^6^Department of Psychiatry, National Taiwan University Hospital, College of Medicine, National Taiwan UniversityTaipei, Taiwan; ^7^Department of Neurology, National Taiwan University Hospital, College of Medicine, National Taiwan UniversityTaipei, Taiwan; ^8^Graduate Institute of Brain and Mind Sciences, College of Medicine, National Taiwan UniversityTaipei, Taiwan; ^9^Department of Psychology, National Taiwan UniversityTaipei, Taiwan; ^10^Graduate Institute of Biomedical Engineering and Bioinformatics, National Taiwan UniversityTaipei, Taiwan

**Keywords:** β-amyloids, Down syndrome, behavioral and psychological symptoms of dementia, Alzheimer’s disease, dementia, tau protein

## Abstract

**Background**: Changes in β-amyloids (Aβ) and tau proteins have been noted in patients with Alzheimer’s disease (AD) and patients with both Down syndrome (DS) and AD. However, reports of changes in the early stage of regression, such as behavioral and psychological symptoms of dementia (BPSD), in DS are sparse.

**Methods**: Seventy-eight controls, 62 patients with AD, 35 with DS and 16 with DS with degeneration (DS_D), including 9 with BPSD and 7 with dementia, were enrolled. The levels of β-amyloids 40 and 42 (Aβ-40, Aβ-42) and tau protein in the blood were analyzed using immunomagnetic reduction (IMR). The Adaptive Behavior Dementia Questionnaire (ABDQ) was used to evaluate the clinical status of the degeneration.

**Results**: The Aβ-40 and tau levels were higher and the Aβ-42 level and Aβ-42/Aβ-40 ratio were lower in DS than in the controls (all *p* < 0.001). Decreased Aβ-40 and increased Aβ-42 levels and Aβ-42/40 ratios were observed in DS_D compared with DS without degeneration (all *p* < 0.001). The ABDQ score was negatively correlated with the Aβ-40 level (*ρ* = −0.556) and the tau protein level (*ρ* = −0.410) and positively associated with the Aβ-42 level (*ρ* = 0.621) and the Aβ-42/40 ratio (*ρ* = 0.544; all *p* < 0.05).

**Conclusions**: The Aβ-40 and Aβ-42 levels and the Aβ-42/Aβ-40 ratio are considered possible biomarkers for the early detection of degeneration in DS. The elevated Aβ-40 and tau levels in DS may indicate early neurodegeneration. The increased Aβ-42 in DS_D may reflect the neurotoxicity of Aβ-42. The paradox of the tau decreases in DS_D could be explained by a burnout phenomenon during long-term neurodegeneration. The different patterns of the plasma beta amyloids and tau protein may imply a different pathogenesis between DS with degeneration and AD in the general population, in spite of their common key pathological features.

## Introduction

Down syndrome (DS), or trisomy 21, is the most common aneuploidy associated with mental retardation, with an incidence of approximately 1 in 800 (Lin et al., 1991; Nussbaum et al., 2001; Centers for Disease Control and Prevention (CDC), 2006). In addition to characteristic facial features, hypotonia, congenital heart disease, gastrointestinal anomalies and learning disabilities, people with DS over 35 years old often suffer from a general acceleration of the aging process, including premature menopause, presbycusis, alopecia, premature graying of the hair, dementia, congestive heart failure, atherosclerosis, diabetes, hypercholesterolemia and hypertension. The close link between the DS phenotype and an increased risk of developing dementia has been addressed previously (Bush and Beail, 2004). The incidence of dementia among DS patients is 8% in the age range of 35–49 years, 55% in the age range 50–59 years and 75% above the age of 60 years (Zana et al., 2007). Owing to the extra copy of chromosome 21, several of the 500 genes on chromosome 21 are overexpressed (Mao et al., 2005), including the *APP* gene, which encodes the amyloid beta (A4) precursor protein (APP).

APP is a membranous protein cleaved by different secretases to form smaller fragments (peptides), some of which are released outside the cells (Zheng and Koo, 2011; Kandimalla et al., 2012). Aβ-40 and Aβ-42 constitute the majority of the released amyloid beta peptides and are produced through a combination of sequential β and γ secretase activities (Selkoe, 1994). Thus, the presence of an extra copy of the* APP* gene leads to the overproduction of Aβ peptides in the brain and peripheral blood in DS (Granic et al., 2010). In Alzheimer’s disease (AD), elevations of Aβ-42 and tau are observed, and increased Aβ-40 in the plasma has been described as one of the biomarkers for AD. In DS, the amyloid plasma levels of Aβ-40 and Aβ-42 are higher than those of age-matched controls (Coppus et al., 2012). A higher Aβ-42 level was proposed to be related to the earlier onset of dementia and mortality, although other studies suggested that a higher Aβ-40 level at baseline predicted the development of dementia. Although the findings are not consistent between studies, they still highlight the importance of Aβ-40 and Aβ-42 in the pathogenesis of dementia in people with DS (Head et al., 2016).

While the deposition of amyloid beta (Aβ) plaques has been observed in young DS individuals, senile plaques and neurofibrillary tangles, the pathological hallmarks of AD, are usually present in DS individuals by the age of 40 (Krasuski et al., 2002; Zana et al., 2007). It has been well documented that the total tau and phosphorylated tau (p-tau 181 and p-tau 231) in the cerebrospinal fluid (CSF) are the main biomarkers for monitoring cortical axonal degeneration in AD (Hampel et al., 2010; Kandimalla et al., 2013; Head et al., 2016). Recently, the concentration of degradation fragments of tau (Tau-A/Tau-C) in the blood was found to be related to the change in cognitive function in early AD patients. In DS with dementia, decreased CSF Aβ-42 and increased tau have been observed (Tapiola et al., 2001).

Recently, the behavioral and psychological symptoms of dementia (BPSD) in DS have been considered as early indicators of clinical dementia (Dekker et al., 2015). However, the diagnosis of BPSD is challenging in DS because of the underlying intellectual disabilities, and the assessment of BPSD in DS has not been well studied (Dekker et al., 2015; Prasher et al., 2015). We have developed a new technology called immunomagnetic reduction (IMR), which is capable of assaying molecules at very low concentrations (Yang et al., 2011; Chiu et al., 2012, 2013, 2014). IMR measures the reduction in the alternating-current magnetic susceptibility of suspended bio-magnetic nanoparticles owing to the immunospecific conjugation between the nanoparticles and Aβ peptides and tau proteins. The Aβ-42 to Aβ-40 concentration ratio and the tau levels in the blood are promising parameters or surrogate biomarkers for evaluating the risk of AD in the general population (Chiu et al., 2012, 2014). In this study, ultra-sensitive IMR was used to assay the plasma Aβ-40, Aβ-42 and tau levels in DS patients. This assay should help us delineate the abnormal concentrations of Aβ-40, Aβ-42 and tau in the blood, which should facilitate the diagnosis of BPSD and dementia in DS. We sought to evaluate the blood amyloid peptides and tau protein in subjects with DS and DS with degeneration (both BPSD and dementia) and to compare these patients with the subjects with AD. The purposes of this study were to explore the changes in blood amyloid and tau in DS and to detect the early deterioration in DS, which could be suggestive of the pathological changes of AD.

## Materials and Methods

### Participants

This study was approved by the Research Ethics Committee of National Taiwan University Hospital (No. IRB No. 201304054RIND, and 201008015R). DS patients who, along with their guardians, agreed to participate were enrolled. Informed consent was signed by the participants and/or their proxy prior to study inclusion in accordance with the Declaration of Helsinki. Participants with DS (*n* = 51) were recruited from the Taiwan DS Foundation and the outpatient clinic of the National Taiwan University Hospital from August 2010 to December 2015. The diagnosis of DS was made by karyotype analysis when patients were born with typical clinical presentations. The mean age of blood sampling was 29.0 ± 10.6 years (range 13–52 years). The subjects were asked to provide a 10-ml venous blood sample. Each sample was assigned a registration number following the sampling sequence so that the laboratory operators were blind to the clinical status and the demographic data of the subjects. The blood samples were centrifuged (2500× g for 15 min) within 1 h of collection, and the supernatant was aliquoted into cryotubes and stored at −80°C until thawed for measurement.

We also included 78 healthy controls (HC) and 62 patients with AD for comparison. In these groups, the assessments performed in this study included determining the clinical dementia rating (CDR) score and the score on the short-form geriatric depression scale (GDS-S); additionally, the Taiwanese mental status examination (TMSE) and two subtests of the Wechsler memory scale (WMS)-III logical memory subtest and family picture subtest were performed (Hua et al., 2005). For the HCs, the cognitive assessments of the participants had to be within normal limits (scores higher than 1.5 standard deviation) of age- and education-matched normal subjects and CDR = 0. For the patients with AD, they must meet the diagnostic guidelines for dementia due to AD proposed by the NIA-AA workgroups in 2011 (Jack et al., 2011; McKhann et al., 2011).

### Clinical Assessment for Regression

The Adaptive Behavior Dementia Questionnaire (ABDQ) was used to evaluate the status of regression of the DS participants (Prasher et al., 2004). The diagnosis of BPSD was made if the patient had the clinical presentation of apathy, agitation, hyperactivity and/or general slowness, depression and psychotic symptoms (Dekker et al., 2015). Dementia was diagnosed by an ABDQ score higher than 78 or by a clinical AD diagnosis according to the degree of cognitive and functional decline; the clinical diagnosis was performed by an adult neurologist (MJC) who is an expert in the diagnosis and management of dementia. DS patients were subgrouped into degeneration (DS_D) if they were diagnosed as having BPSD or dementia. The CDR was used to score non-DS populations for AD as a comparison.

### Immunomagnetic Reduction Assay

Aβ-40, Aβ-42 and tau were assayed by IMR with a high-temperature superconducting quantum interference device (SQUID) magnetometer, as previously reported. In brief, 3 reagents (MF-AB0-0060, MF-AB2-0060 and MF-TAU-0060, MagQu, New Taipei City, Taiwan) were used to assay the biomarkers Aβ-40, Aβ-42 and tau, respectively. These reagents were then mixed with individual samples. Each mixture was placed in a SQUID-based alternating-current (ac) magnetic susceptometer (XacPro-S, MagQu, Taiwan), and the IMR signals from these samples were detected and converted to the concentrations of Aβ-40, Aβ-42 and tau using the characteristic curves. For detailed procedures and technical information, please refer to our previous reports.

### Statistical Analysis

Data were expressed as the mean ± standard deviation. The Mann-Whitney U test was used. Spearman’s rank correlation coefficient was used to determine the correlations between the ABDQ score and the Aβ-42/Aβ-40 ratio and Aβ-40, Aβ-42 and tau levels. *P* < 0.05 was considered statistically significant. SPSS (SPSS, Inc., 2008, SPSS Statistics for Windows, Version 17.0. Chicago, IL, USA) was used to conduct receiver operating characteristic (ROC) curve analysis. The optimal threshold for the ROC curve was determined by Youden’s index using the maximum summation of the sensitivity plus specificity with respect to DS without degeneration as a control (Youden, 1950).

## Results

### Elevation of Baseline Aβ-40 Levels in DS Patients Compared to Normal Subjects

**Figure 1:**
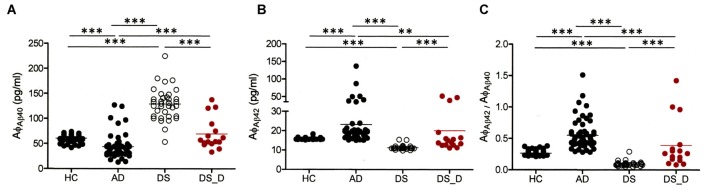
**β-amyloids 40 (Aβ-40; Aφ_Aβ40_) and Aβ-42 (Aφ_Aβ42_) levels and the Aβ-42/Aβ-40 (Aφ_Aβ42_/Aφ_Aβ40_) ratio in healthy controls (HC, combining the younger and older groups) and in patients with Alzheimer’s disease (AD), Down syndrome (DS) and DS with degeneration (DS_D; behavioral and psychological symptoms of dementia (BPSD) and dementia)**. **(A)** For Aβ-40, *p* < 0.001 between HC and AD; *p* < 0.001 between HC and DS; *p* < 0.001 between AD and DS; *p* = 0.497 between HC and DS_D; *p* < 0.001 between AD and DS_D; and *p* < 0.001 between DS and DS_D. **(B)** For Aβ-42, *p* < 0.001 between HC and AD; *p* < 0.001 between HC and DS; *p* < 0.001 between AD and DS; *p* = 0.070 between HC and DS_D; *p* = 0.001 between AD and DS_D; and *p* < 0.001 between DS and DS_D. **(C)** For Aβ-42/Aβ-40, *p* < 0.001 between HC and AD; *p* < 0.001 between HC and DS; *p* < 0.001 between AD and DS_D; *p* = 0.888 between HC and DS_D; *p* < 0.001 between AD and DS; and *p* < 0.001 between DS and DS_D. **p < 0.01; ***p < 0.001.

The demographic data of the participants are listed in Table 1. We first divided the HC into younger and older groups. Because the biomarker levels did not show significant differences, we combined them into one group (HC). The Aβ-40 level was higher in DS patients than in healthy subjects (both younger and older groups), (Table 1, Figure 1A; *p* < 0.001). However, the Aβ-42 level and the Aβ-42/Aβ-40 ratio were lower in the DS patients than in the HC (Figures 1B,C; both *p* < 0.001). Compared to the HC, individuals with non-DS AD had a lower Aβ-40 level and a higher Aβ-42 level and Aβ-42/Aβ-40 ratio (*p* < 0.01, *p* = 0.01 and *p* < 0.01, respectively). This pattern was also observed in DS subjects. Compared to those with DS without degeneration, the patients with DS with degeneration (DS_D; BPSD and dementia) had a lower Aβ-40 level and a higher Aβ-42 level and Aβ-42/Aβ-40 ratio (all *p* < 0.001).

**Table 1:** **Demographic and biomarker data of the participants**.

Group	HC_Y	HC_E	AD	DS	DS_D
Number	44	34	62	35	16
Female/Male	14/30	20/14	32/29	16/19	6/10
Age (years)	38.4 ± 13.5	70.3 ± 5.8	72.1 ± 11.1	25.6 ± 9.3	35.4 ± 10.7
(range)	(23–59)	(61–81)	(42–93)	(13–51)	(20–52)
ABDQ_DS_	−	−	−	25.4 ± 13.8	58.8 ± 20.8
CDR_AD (n)_	0	0	31/19/9/3	−	−
Aβ-40 (pg/ml)	61.1 ± 6.3	60.7 ± 6.9	43.9 ± 22.1	129.0 ± 31.8	69.5 ± 31.5
Aβ-42 (pg/ml)	15.8 ± 0.3	16.0 ± 0.5	23.2 ± 18.4	11.3 ± 1.1	20.1 ± 13.2
Aβ-42/Aβ-40	0.26 ± 0.03	0.27 ± 0.04	0.55 ± 0.23	0.10 ± 0.04	0.39 ± 0.38
Tau (pg/ml)	14.9 ± 5.5	15.0 ± 7.3	47.5 ± 18.9	31.5 ± 8.0	26.3 ± 17.3

*HC-Y, young healthy controls; HC-E, older healthy controls; DS, Down syndrome; AD, Alzheimer’s disease; DS_D, DS with degeneration (behavioral and psychological symptoms of dementia (BPSD) or dementia); ABDQ, Adaptive Behavior Dementia Questionnaire; CDR, clinical dementia rating 0.5/1/2/3*.

### Correlation Between ABDQ Score and Aβ Results

**Figure 2:**
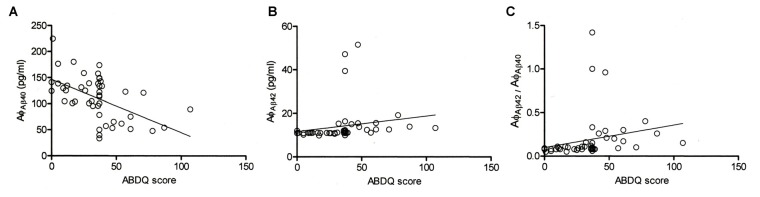
**Correlation of the Adaptive Behavior Dementia Questionnaire (ABDQ) score with the Aβ-40 (Aφ_Aβ40_) and Aβ-42 (Aφ_Aβ42_) levels and the Aβ-42/Aβ-40 (Aφ_Aβ42_/Aφ_Aβ40_) ratio in DS patients (combined DS and DS_D groups)**. A significant negative correlation between **(A)** the Aβ-40 level and ABDQ score (*ρ* = −0.556; *p* < 0.001) and **(B)** positive associations between the ABDQ score and the Aβ-42 level (*ρ* =0.621; *p* < 0.001) and **(C)** Aβ-42/Aβ-40 ratio (*ρ* = 0.544; *p* < 0.001) were noted.

ABDQ has been used as a screening tool for dementia in DS. In our subjects with DS, there were significant differences in the ABDQ score between subjects with and without degeneration (BPSD and dementia; Table 1; 60.6 ± 19.8 vs. 25.4 ± 13.8; *p* < 0.001). No significant difference was found in the ABDQ scores between patients with DS with dementia and those with DS with BPSD (71.8 ± 24.3 vs. 52.1 ± 10.9; *p* = 0.080). We found a significant negative correlation between the Aβ-40 level and ABDQ score (*ρ* = −0.556; *p* < 0.001) and positive associations between the Aβ-42 level (*ρ* = 0.621), the Aβ-42/Aβ-40 ratio (*ρ* = 0.544) and the ABDQ score (both *p* < 0.001; Figure 2).

### Prediction of Degeneration Using Amyloid

**Figure 3:**
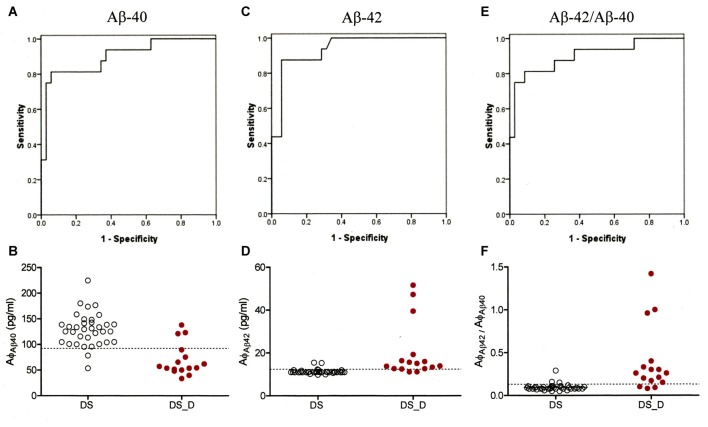
**Receiver operating characteristic (ROC) curve of Aβ-40, Aβ-42 and Aβ-42/Aβ-40 for the differential diagnosis of DS without (DS) and with degeneration (DS_D). (A)** ROC curve of Aβ-40. **(B)** Concentration of Aβ-40 for the differential diagnosis between DS and DS_D. **(C)** ROC curve of Aβ-42. **(D)** Concentration of Aβ-42 for the differential diagnosis between DS and DS_D. **(E)** ROC curve of Aβ-42/Aβ-40. **(F)** Concentration of Aβ-42 for the differential diagnosis between DS and DS_D.

To characterize the diagnostic possibilities, we performed ROC curve analysis to compare the patients with DS and those with DS_D (Figure 3). The areas under the curve (AUCs) of the Aβ-40 and Aβ-42 levels and the Aβ-42/Aβ-40 ratio were 0.900, 0.937 and 0.902, respectively. The sensitivity and specificity in differentiating DS from DS_D were 81.3% and 94.3%, respectively, using Aβ-40, with a threshold of 92.2 pg/ml (Figures 3A,B); 87.5% and 94.3%, respectively, using Aβ-42, with a threshold of 12.36 pg/ml (Figures 3C,D); and 81.3% and 91.4%, respectively, using the Aβ-42/Aβ-40 ratio, with a threshold of 0.13 (Figures 3E,F).

### Elevation of Baseline Tau Levels in DS Patients Compared to Normal Subjects

**Figure 4:**
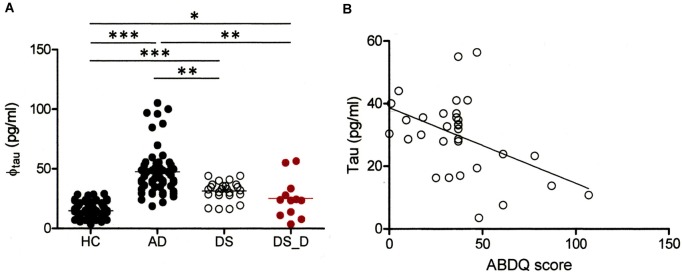
**Comparison of tau protein in different groups. (A)** Tau protein (φ_tau_) in HC and in subjects with AD, DS or DS with degeneration (BPSD and with dementia; DS_D). For tau, *p* < 0.001 between HC and AD; *p* < 0.001 between HC and DS; *p* < 0.001 between AD and DS; *p* = 0.019 between HC and DS_D; *p* = 0.001 between AD and DS_D; and *p* = 0.140 between DS and DS_D. *p < 0.05; **p < 0.01; ***p < 0.001. **(B)** Correlation of the Adaptive Behavior Dementia Questionnaire (ABDQ) score with the tau. A significant negative correlation between the Aβ-40 level and ABDQ score was noted (*ρ* = −0.410; *p* = 0.018).

We also determined the tau concentration in 21 DS participants, including 9 with DS_D. In the DS subjects, the baseline tau protein level was higher than that in the HC (Table 1 and Figure 4A; 31.5 ± 8.0 vs. 14.9 ± 5.5 pg/ml; *p* < 0.001). We found a significant negative correlation between tau and the ABDQ score in DS patients (combine DS and DS_D groups; Figure 4B, *ρ* = −0.410; *p* = 0.018). This finding supports the relationship between cognitive/functional declines and tau profile changes. However, the ROC curve analysis for differentiating DS from DS_D using tau yielded an AUC of only 0.49.

## Discussion

Because of the baseline cognitive impairment in DS subjects, it is challenging to diagnose dementia and detect early BPSD in DS populations. Several assessment tools and questionnaires can be used to evaluate the signs and symptoms of AD in DS, including ABDQ, the Dementia Scale for Down Syndrome (DSDS), the Dementia Screening Questionnaire for Individuals with Intellectual Disabilities (DSQIID), the Dementia Questionnaire for Mentally Retarded Persons (DMR) and the recently developed Rapid Assessment for Developmental Disabilities (RADD). However, these tools are not satisfactory because of their ceiling or floor effects. In this study, we not only used ABDQ to screen DS patients but also employed a neurologist who specializes in the diagnosis and management of patients with dementia to evaluate the general behavior presentation for the diagnosis of AD in DS subjects.

In this study, we demonstrated that compared with healthy subjects in general, adolescent DS subjects already had elevated levels of Aβ-40 and tau protein. The baseline elevation of Aβ-40 in DS adolescents and adults is a reasonable finding based on the APP protein overexpression in DS, with the majority of Aβ cleaved into Aβ-40. This finding is consistent with previous reports (Schupf et al., 2001, 2010; Head et al., 2011; Coppus et al., 2012). Furthermore, this result suggests that APP is abnormally cleaved in DS subjects with dementia and indicates that the underlying pathophysiology of DS with dementia might be different from that of non-DS patients with AD.

We also found that the Aβ-40 is decreased and the Aβ-42 and Aβ-42/Aβ-40 ratio are increased in the blood of DS_D patients. This observation may imply that with degeneration, a higher percentage of APP is cleaved into Aβ-42 rather than Aβ-40. The finding of increases in the Aβ-42 level and Aβ-42/Aβ-40 ratio in DS_D patients is in agreement with previous reports of observations of non-DS populations with mild cognitive impairment and AD. In the DS subjects, the majority of reports demonstrated higher Aβ-42 levels at the baseline compared with those of HC and a higher Aβ-42 level and Aβ-42/Aβ-40 ratio in those with DS with dementia. This finding further suggests that Aβ-42 is a critically toxic substance to the brain in both groups of subjects with AD, with or without DS. In addition, we demonstrated a negative correlation between the ABDQ score and the Aβ-40 level and a positive association between the ABDQ score and the Aβ-42/Aβ-40 ratio in DS subjects. This correlation may imply their possible utility as biomarkers to help evaluate the degeneration in DS.

The tau protein is a microtubule-associated protein located inside neurons, and hyperphosphorylated tau is the main component of the neurofibrillary tangles in AD. Because tau is an intracellular protein, a higher baseline tau level in DS might indicate early neurodegeneration (neuronal damage), and tau may consequently be elevated in DS subjects before the onset of dementia or BPSD. However, we found a negative correlation between the tau protein and ABDQ and observed a lower tau level in DS_D. This paradox may be explained by a possible burnout phenomenon of neurons during the prolonged neurodegenerative process and a consequently lower level of tau. A longitudinal study is required to confirm our proposed explanation.

In this study, we found that the Aβ-40 and Aβ-42 levels and the Aβ-42/Aβ-40 ratio may be good indicators for the early detection of degeneration in DS. In our previous reports in a general population with mild cognitive impairment and AD, the differential sensitivity and specificity of Aβ-42 and tau between HC and non-DS AD were approximately 0.9 (0.88–0.97), using thresholds of 16.33 pg/ml for Aβ-42 and 23.89 pg/ml for tau. The threshold for Aβ-42 that we used to differentiate DS_D from DS was 12.36 pg/ml, with a sensitivity of 0.875 and a specificity of 0.943, which was lower than that for non-DS AD. The reason is not clear but could be the younger age of the DS cohort and the inclusion of DS with BPSD, which probably represents an early stage of DS AD. The threshold for tau was 26.04 pg/ml, with a sensitivity of 0.636 and specificity of 0.8883; this threshold was higher than that for non-DS AD. However, in DS, the baseline tau level was higher than in the general population, and a decrease in tau was noted in DS with AD. These results indicate that the tau levels that demarcate AD differ between DS AD and non-DS AD.

This study has some limitations. First, the mean age of our DS subjects was younger than that of the HC because of the difficulty of obtaining blood samples from adolescents for ethical reasons in our hospital. We tried to divide the HC into two different age groups (ages of 23–59 years and 61–81 years), but the amyloid and tau levels did not exhibit significant between-group differences. Therefore, we pooled the results of the healthy subjects from both age groups into one single group for further analysis to improve the statistical power. However, this gap could be overcome by evidence from previous reports that DS subjects have elevated plasma Aβ-40 levels compared to those of age-matched HC (Schupf et al., 2001, 2010; Head et al., 2011; Coppus et al., 2012). Second, the small sample size of the subjects of DS with dementia, especially the number of tau samples, hindered the statistical analysis. Third, we did not analyze other risk factors for dementia, such as *APP* or presenilin (*PSEN1* and *PSEN2*) mutations, although the prevalence of these factors in dementia is extremely low in the general population. Other possible contributing factors for dementia should be explored in DS subjects in future studies. Finally, recent studies revealed that the elevation of tau in the blood is related to changes in the cognitive function over time in early AD subjects. In contrast, we observed a decline in tau levels in DS with degeneration (which were still higher than those of HC) and proposed a burnout phenomenon of brain tissue from the prolonged neurodegeneration process. A longitudinal cohort study is required to confirm the finding of the tau decline during the process of DS degeneration.

## Conclusion

The diagnosis of dementia and/or BPSD in DS is challenging. This study establishes the capability of differentiating between DS with and without degeneration. The beta amyloid and tau levels and the ABDQ score may assist in the early detection of regression in DS, which might represent an early Alzheimer’s pathology. However, the different patents of the plasma beta amyloids and tau protein may imply a different pathogenesis between DS with degeneration and AD in the general population, in spite of their common key pathological features.

## Author Contributions

N-CL and M-JC conducted the study and performed the statistical analysis and main drafting of the manuscript. S-YY and J-JC performed the SQUID analysis. P-TH, L-MC, A-CH and Y-NC performed the behavior evaluations and participated in the design and coordination of the study. Y-HC and W-LH jointly conceived the study. All authors read and approved the final manuscript.

## Funding

This work was supported in part by the Ministry of Science and Technology of Taiwan 102-2314-B-002-085, 104-2745-B-003-002 and 102-2923-B-002-007-MY3; and the Ministry of Economic Affairs of Taiwan under grant numbers 101-EC-17-A-17-I1-0074, 1Z970688 and 1Z0990415 (SBIR).

## Conflict of Interest Statement

The authors declare that they have no competing interests.

## Abbreviations

Aβ: amyloid beta
ABDQ: Adaptive Behavior Dementia Questionnaire
AD: Alzheimer’s disease
APP: amyloid beta (A4) precursor protein
BPSD: behavioral and psychological symptoms of dementia
CDR: clinical dementia rating
CSF: cerebrospinal fluid
DMR: Dementia Questionnaire for Mentally Retarded Persons
DS: Down syndrome
DS_D: Down syndrome with degeneration
DSDS: Dementia Scale for Down Syndrome
DSQIID: Dementia Screening Questionnaire for Individuals with Intellectual Disabilities
IMR: immunomagnetic reduction
RADD: Rapid Assessment for Developmental Disabilities
ROC: receiver operating characteristic
SQUID: superconducting quantum interference device.
